# Treatment options for subjective tinnitus: Self reports from a sample of general practitioners and ENT physicians within Europe and the USA

**DOI:** 10.1186/1472-6963-11-302

**Published:** 2011-11-04

**Authors:** Deborah A Hall, Miguel JA Láinez, Craig W Newman, Tanit Ganz Sanchez, Martin Egler, Frank Tennigkeit, Marco Koch, Berthold Langguth

**Affiliations:** 1 NIHR National Biomedical Research Unit in Hearing, Nottingham, UK; 2 Division of Psychology, School of Social Sciences, Nottingham Trent University, Nottingham, UK; 3 Department of Neurology, University Clinic Hospital, University of Valencia, Valencia, Spain; 4 Head and Neck Institute, Cleveland Clinic, Cleveland, Ohio, USA; 5 Department of Otorhinolaryngology, University of São Paulo School of Medicine, São Paulo, Brazil; 6 Merz Pharmaceuticals GmbH, Frankfurt Am Main, Germany; 7 Department of Psychiatry and Psychotherapy, University of Regensburg, Regensburg, Germany

## Abstract

**Background:**

Tinnitus affects about 10-15% of the general population and risks for developing tinnitus are rising through increased exposure to leisure noise through listening to personal music players at high volume. The disorder has a considerable heterogeneity and so no single mechanism is likely to explain the presence of tinnitus in all those affected. As such there is no standardized management pathway nor singly effective treatment for the condition. Choice of clinical intervention is a multi-factorial decision based on many factors, including assessment of patient needs and the healthcare context. The present research surveyed clinicians working in six Westernized countries with the aims: a) to establish the range of referral pathways, b) to evaluate the typical treatment options for categories of subjective tinnitus defined as acute or chronic, and c) to seek clinical opinion about levels of satisfaction with current standards of practice.

**Methods:**

A structured online questionnaire was conducted with 712 physicians who reported seeing at least one tinnitus patients in the previous three months. They were 370 general practitioners (GPs) and 365 ear-nose-throat specialists (ENTs) from the US, Germany, UK, France, Italy and Spain.

**Results:**

Our international comparison of health systems for tinnitus revealed that although the characteristics of tinnitus appeared broadly similar across countries, the patient's experience of clinical services differed widely. GPs and ENTs were always involved in referral and management to some degree, but multi-disciplinary teams engaged either neurology (Germany, Italy and Spain) or audiology (UK and US) professionals. For acute subjective tinnitus, pharmacological prescriptions were common, while audiological and psychological approaches were more typical for chronic subjective tinnitus; with several specific treatment options being highly country specific. All therapy options were associated with low levels of satisfaction.

**Conclusions:**

Despite a large variety of treatment options, the low success rates of tinnitus therapy lead to frustration of physicians and patients alike. For subjective tinnitus in particular, effective therapeutic options with guidelines about key diagnostic criteria are urgently needed.

## Background

Tinnitus is defined as a perceived noise of varying intensity, loudness and pitch in the absence of an external sound [1,2] Although the experience of short bursts of noise is almost universal, tinnitus is typically defined as noise that lasts at least 5 minutes [3]. Tinnitus can be either acute or chronic. In the present study, chronic tinnitus is defined as a condition lasting longer than three months. Its prevalence is reported to be about 10 to 15% of the general population, but it is more common in adults, especially older adults [4]. For example, 12% of over 60 year olds, but only 5% of 20 to 30 year olds are reported to experience chronic tinnitus [5].

Tinnitus is described as subjective or objective. While objective tinnitus has a physical explanation for the perceived sound, subjective tinnitus can be heard only by the sufferer (i.e. a 'phantom sensation'). Objective tinnitus is much less common in the clinic. Subjective tinnitus is therefore the main topic of this publication.

Among severe sufferers, tinnitus causes disability associated with concentration deficits, insomnia, hypersensitivity to sounds, anxiety and depression. Often a combination of several complaints leads to a diminished quality of life [6,7] For example, it is estimated that for about 1 in 100 of the general population, the condition severely affects their quality of life [2]. Emotional distress is not simply related to tinnitus loudness; it depends furthermore on whether the tinnitus is perceived as threatening [8]. In this respect, tinnitus can be described as 'compensated' (i.e. the patient notices the noise in his/her ear or head, but it does not impair quality of life) or 'not compensated' (i.e. a severe condition that severely impacts on quality of life, with the possible development of secondary symptoms such as anxiety).

### Pathophysiology

It has been estimated that 85% of tinnitus cases are accompanied by hearing loss and that occupational and leisure noise are the greatest factors causing cochlear damage [9]. Clinically, tinnitus is not a unitary condition and its aetiology has also been associated with head and neck injuries, ototoxic drugs, vascular and cerebrovascular diseases, systemic disorders, infectious disease, autoimmune disorders, ear conditions and temporo-mandibular joint disorders [10]. Despite these various causes, it is now well established that the central auditory system plays an important role in the perception of tinnitus [11]. Recent research showed that changes in neuronal activity might underlie tinnitus pathology, but our knowledge of the precise neural substrates of tinnitus is still limited also because studies have difficulties to separate what changes have been induced by hearing loss and what are specifically associated with tinnitus [1,12].

### Treatment options

Currently there is no cure for tinnitus [13], nor are there any licensed medications for alleviating the symptoms. For example, there is no Food and Drug Administration (FDA) or European approved drug specifically for treating subjective tinnitus [14]. Reasons that hamper the identification of good candidates for an effective pharmacological treatment for tinnitus include the heterogeneity of tinnitus and our limited knowledge about the pathophysiology of the different forms of tinnitus. Consequently, a majority of tinnitus treatment options are primarily directed towards alleviating or managing the accompanying symptoms making the tinnitus less intrusive or less distressing.

Several comprehensive reviews of the therapeutic options are provided in the literature [11,15]. Most common approaches include education, acoustic devices (hearing aids, noise generators, combination devices), psychological therapy (e.g. cognitive-behavioural therapy, counselling and relaxation), Tinnitus Retraining Therapy (TRT), and complementary treatments such as acupuncture. Acoustic devices enrich the sound environment and can thus be helpful in diminishing patients' awareness of their tinnitus, while a majority of clinical 'packages' involve psychological and/or educational components aimed at changing the emotional meaning of the tinnitus in order to reduce personal distress [16]. A good source of evidence for the efficacy of the various treatment options is collated in the Cochrane Collection http://www2.cochrane.org/reviews which provides major reviews of tinnitus-related publications. However, examination of this resource indicates rather contradictory or poor-quality evidence for a number of strategies used in current practice.

Pharmacological treatments are also widely available for reducing the various co-morbid symptoms such as depression and insomnia. The most frequently used drug options were recently summarized by Langguth and colleagues [14]. Due to the comorbidity of depression in some chronic tinnitus patients, antidepressants are commonly used. Antidepressants may be able to alleviate sleep problems and other depressive symptoms for some severely distressed patients. It has been recommended that they should be used in combination with psychological therapy for the most effective management of tinnitus [15]. Other drugs include tranquillisers (especially benzodiazepines), anti-vertigo products (e.g. cinnarizine), coronary drugs (especially niacin), local anaesthetics (e.g. lidocaine), or natural remedies/supplements (e.g. bioflavonoids, zinc). In Germany, a local preference has been rheological infusion which is thought to improve the rate of blood flow, but reimbursement for this type of treatment ceased in 2009. Again, the efficacy of these drugs in relieving the symptoms of tinnitus is uncertain.

### Clinical practices

While the number of different treatment options for tinnitus is growing, very few studies have examined the extent to which this has impacted on clinical practice. Of all the different specialties that contribute to tinnitus referral and management, general practitioners (GPs) and otolaryngology specialists (ENTs) play substantial roles in all six countries that were surveyed. Our survey therefore targets these two professions. From their postal survey of general practitioners (GPs) in the UK, Vanniasegaram and colleagues [17] concluded that there was a substantial discrepancy between the scientific and technological perspectives on the management of tinnitus and the *actual *day-to-day practice in the primary care setting. This situation seems to have changed little almost 20 years on. A national postal survey of GPs in the UK conducted by El-Shunnar and colleagues [18] highlighted similar findings. Although some GPs highlighted little demand for tinnitus management within their practice, many others expressed an unmet need for specific and concise training on tinnitus management.

Patients themselves also express dissatisfaction. For example, a recent telephone survey of members of the Irish Tinnitus Association revealed that many healthcare professionals (especially GPs) appeared to have limited awareness of developments in the therapeutic field and provided little advice or information to help people manage their condition [19]. This finding was recently echoed in a survey conducted in Northern Ireland by the Royal National Institute for Deaf People (RNID) [20]. One way to address the problem would be to include tinnitus management in the training curriculum for medical students and/or GPs [17,20]. To our knowledge this has not been taken up and so alternatively protocols (flowcharts) for diagnosis and treatment could be made available. First efforts towards the development of flowchart for diagnosis and management have been initiated [21,22], but a widely accepted protocol for how to diagnose and refer or select an appropriate treatment option has yet to be established. Hoare and Hall [23] have recently argued that some degree of standardization in practice is essential for identifying key standards of best practice for tinnitus, for ensuring equal patient access to treatments, for facilitating clinical audit, and for providing high-level evidence of clinical efficacy. Several organisations have recently introduced standardized protocols, developed from broad collaborative efforts. In the UK, the Good Practice Guide (GPG) document produced by the Department of Health is targeted at managers in the National Health Service [21].

The Tinnitus Research Initiative (TRI) more recently produced a document targeted at an international audience of hearing practitioners and medical specialists [22]. Both protocols address diagnostic assessment and treatment, but the TRI document focuses heavily on a medical model of tinnitus management, while the GPG is more representative of a patient-centred approach. In the UK at least, the influence of the GPG has been somewhat limited [24] and on the international front perhaps it is too premature to expect any impact from the TRI document. Today it would seem that the choice of treatment remains largely in the hands of the individual clinical professional, perhaps influenced also by country-specific training routes and practices, schemes for reimbursement from medical insurance, other local resource limitations and patient preference.

With this survey, we aim to shed some light on the way physicians working in different countries report handling acute and chronic tinnitus and to find their preferred modes of treatment. Finding possible country-specific preferences in treating tinnitus patients may help to uncover possible weak points in patient care and medical alternatives, and to find a more standardized approach of treatment. We use an online questionnaire administered to a sample of physicians to explore number of tinnitus patients consulted in the past three months, pathogenesis (subjective vs objective), duration (acute vs chronic), severity (compensated vs not compensated), referral, responsibility for diagnosis and management, *normal *treatment options and their perceived success rate of treatment. A number of questions specifically asked about the range of pharmaceutical treatments used as first and second line approaches, and satisfaction with those medications. In particular, we aim to highlight differences between the two clinical professions and also between clinical practices in six countries. Our discussion seeks to contextualize some of these findings into the specific healthcare system operating within the different countries.

## Methods

### Questionnaire

The online survey was performed by DocCheck Medical Services GmbH for Merz Pharmaceuticals GmbH between February 6^th ^and March 2^nd ^2007. The questionnaire consisted of 16 questions requiring a range of numerical responses, self ratings and open text responses, including self-report estimates of the number of tinnitus patients consulted in the past 3 months, pathogenesis, duration, severity, referral, diagnosis, management, success rate, and satisfaction (Appendix 1). Four of the questions specifically related to pharmaceutical treatments for tinnitus. Questionnaire translation was a multi-step process. A master version was prepared in English and then translated into German, Italian, Spanish and French. A native speaker worked independently to proofread each translation. The questionnaire was scripted with the software Umfragecenter^®^.

### Recruitment approach

The research institute DocCheck, Germany, together with its international partners offers country-wide panels of thousands of physicians from all clinical specialties who are willing to share their expert views in anonymous online market research. Although this is a voluntary sample from those registered in the GP and ENT communities, the demographic distribution of respondents (age, gender, region) is cross-checked against available population statistics for the two professions to provide some assurance about representativeness. Target countries for this survey were the USA and five European countries (UK, Germany, France, Spain and Italy).

Email invitations to the screening interview were sent out to the relevant specialty panels. Physicians qualified to participate in this survey if they were general practitioners/primary care physicians (thereafter denoted as GPs) or otolaryngologists/Ear-Nose-Throat specialists (thereafter denoted as ENTs). From each country, GPs and ENTs were recruited in equal proportions. Approximate quotas were pre-set for each country. In the US, it was 200 and in European countries it was 100. As far as possible, recruitment considered demographic and regional variables.

### Respondents

Overall, 370 GPs and 365 ENTs were surveyed. Seventeen GPs and 6 ENTs (mostly in the US) were screened out because they had not seen any tinnitus patients in the last three months, leaving a total of 712 respondents (Table 1), with an average age of 49 years. Typically, these respondents were male. For GPs, 76% were male (267/353) and for ENTs, 83% were male (297/359).

**Table 1:** Total number of participating physicians and their demographic characteristics.

	Profession	Gross sample [N]	Sample after screening [N]	Male [N]	Female [N]	Average age [years]
US	GPs	117	102	70	32	42
	ENTs	106	102	88	14	48
UK	GPs	52	51	40	11	47
	ENTs	54	53	49	4	46
Germany	GPs	51	50	34	16	50
	ENTs	50	50	36	14	52
France	GPs	50	50	43	7	49
	ENTs	50	50	43	7	51
Spain	GPs	50	50	39	11	49
	ENTs	52	52	39	13	50
Italy	GPs	50	50	41	9	52
	ENTs	53	52	42	10	51

GP = General practitioner, ENT = Ear-Nose-Throat specialist. Screening excluded those physicians who had not seen any tinnitus patients in the past three months. All values represent total numbers (except age, which represents an average).

## Results

### Number of tinnitus patients consulted in the last three months

Table 2 reports the distribution of the number of estimated consultations for tinnitus in the preceding three-month period. From the total number of 735 respondents, the median number of GP consultations was 10-19. In all countries, the distribution for ENTs tended to appear shifted upwards such that the median number of ENT consultations for the same period was 30-49. Germany appeared to be a prominent exception since 70% of responding ENTs estimated that they had more than 100 appointments with tinnitus patients in the three-month period.

**Table 2:** Number of professionals and their estimate of the number of tinnitus consultations in the last three months.

	GP profession						
**# patients consulted**	**US**	**UK**	**Germany**	**France**	**Spain**	**Italy**	**GP total**
0	15	1	1	-	-	-	17
1-4	28	13	3	4	9	8	65
5-9	18	11	11	10	6	12	68
10-19	26	13	14	15	16	17	101
20-29	16	7	6	11	8	6	54
30-49	11	4	11	6	4	6	42
50-99	3	2	5	3	7	1	21
>100	-	1	-	1	-	-	2

	**ENT Profession**						

**# patients consulted**	**US**	**UK**	**Germany**	**France**	**Spain**	**Italy**	**ENT total**
0	4	1	-	-	-	1	6
1-4	3	1	-	1	-	-	5
5-9	2	1	-	-	1	6	10
10-19	13	5	2	9	7	13	49
20-29	18	8	2	8	8	7	51
30-49	21	13	2	14	18	10	78
50-99	26	17	9	10	10	9	81
>100	19	8	35	8	8	7	85
							
							

	**GP profession**						

**# new patients consulted**	**US**	**UK**	**Germany**	**France**	**Spain**	**Italy**	**GP total**
0	20	5	2	-	1	1	29
1-4	45	32	25	23	18	35	178
5-9	22	7	17	17	14	10	87
10-19	25	6	6	10	12	4	63
20-29	5	-	1	-	2	-	8
30-49	-	2	-	-	2	-	4
50-99	-	-	-	-	1	-	1
>100	-	-	-	-	-	-	0

	**ENT profession**						

**# new patients consulted**	**US**	**UK**	**Germany**	**France**	**Spain**	**Italy**	**ENT total**
0	4	1	-	-	-	1	6
1-4	8	3	1	4	2	10	28
5-9	9	1	1	6	9	11	37
10-19	23	11	6	21	18	10	89
20-29	21	12	6	9	12	10	70
30-49	14	13	10	2	6	8	53
50-99	19	10	11	5	4	2	51
>100	8	3	15	3	1	1	31

Data are reported separately across the two professions and each country.

Table 3 reports the estimated numbers of new patients reporting tinnitus. For GPs, the median number was 1-4 new consultations in the past three months. Hence we can infer that a substantial majority of tinnitus consultations are with people who have *repeated *appointments. For ENTs, the median number of new patients was substantially higher than for GPs (20-29). We can infer that the caseload for ENTs is more evenly balanced across new and repeating patients.

**Table 3:** Number of professionals and their estimate of the number of new tinnitus consultations in the last three months.

	GP profession						
**# new patients consulted**	**US**	**UK**	**Germany**	**France**	**Spain**	**Italy**	**GP total**
0	20	5	2	-	1	1	29
1-4	45	32	25	23	18	35	178
5-9	22	7	17	17	14	10	87
10-19	25	6	6	10	12	4	63
20-29	5	-	1	-	2	-	8
30-49	-	2	-	-	2	-	4
50-99	-	-	-	-	1	-	1
>100	-	-	-	-	-	-	0

	**ENT profession**						

**# new patients consulted**	**US**	**UK**	**Germany**	**France**	**Spain**	**Italy**	**ENT total**
0	4	1	-	-	-	1	6
1-4	8	3	1	4	2	10	28
5-9	9	1	1	6	9	11	37
10-19	23	11	6	21	18	10	89
20-29	21	12	6	9	12	10	70
30-49	14	13	10	2	6	8	53
50-99	19	10	11	5	4	2	51
>100	8	3	15	3	1	1	31

Data are reported separately across the two professions and each country.

For the subsequent analyses, we included only those healthcare professionals who reported a consultation with at least one tinnitus patient within the past three months. Hence, 17 GPs and 6 ENTs were excluded (see also Tables 1 and 2). The analysis is primarily presented in the form of descriptive statistics reporting the percentage of responses across each profession. Many of the questions relate to subjective tinnitus and these responses are split by country to paint a picture of the international variations. Typical treatment options, their perceived success rate, and range of pharmacological options were analysed separately for acute and chronic subjective tinnitus.

### Pathogenesis

A small number of physicians indicated a lack of understanding of the terminology ('subjective/objective'). Overall, 13 GPs (US 4; UK 3; France 4; Spain 2) and 3 ENTs (US 1; Spain 1; Italy 1) reported unfamiliarity with this classification. Of those GPs and ENTs remaining, subjective tinnitus was judged to account for about three quarters of cases (65-82% for GPs and 71-88% for ENTs). Table 4 reports the percentage of patients reporting subjective and objective tinnitus across the six countries. Results demonstrate how objective tinnitus represents the minority of tinnitus cases.

**Table 4:** Percentage of patients classified as having subjective or objective tinnitus, split by each country, and reported separately for each professional group.

	GP profession		ENT profession	
**Country**	**Subjective** **[%]**	**Objective** **[%]**	**Subjective** **[%]**	**Objective** **[%]**
				
US	72	28	78	22
UK	75	25	88	12
Germany	82	18	86	14
France	77	23	83	17
Spain	65	35	75	25
Italy	71	29	71	29

Data are reported only for those physicians who reported seeing at least one tinnitus patient in the past three months.

### Duration

Chronic tinnitus (≥ 3 months) was reported to account for 64% of cases overall (Table 5). There were no striking differences in the pattern between GPs and ENTs, nor between the six countries.

**Table 5:** Percentage of patients classified as having chronic (≥ 3 months) or acute (< 3 months) tinnitus, split by each country, and reported separately for each professional group.

	GP profession		ENT profession	
**Country**	**Chronic** **[%]**	**Acute** **[%]**	**Chronic** **[%]**	**Acute** **[%]**
				
US	50	50	69	31
UK	68	32	81	19
Germany	66	34	53	47
France	59	41	71	29
Spain	49	51	72	28
Italy	61	39	66	34

Data are reported only for those physicians who saw at least one tinnitus patient in the past three months.

### Severity

There were no striking differences in the pattern between GPs and ENTs, nor between the six countries (Table 6). Typically, patients with compensated symptoms were slightly in the majority (GPs: 51-66%, ENTs: 58-74%). It is perhaps somewhat surprising to find that patients whose tinnitus does not substantially impact on their quality of life appeared to be the ones most often seeking help. We rule out a lack of understanding of the terminology ('compensated/not compensated') since only 13 GPs (US 9; UK 3; France 1) and 2 ENTs (US 1; France 1) reported unfamiliarity with this classification. It is therefore possible that a substantial number of consultations either just require advice and reassurance about non-bothersome tinnitus or require some treatment which does alleviate associated symptoms, but that those symptoms nonetheless are not judged to impair quality of life. Ear wax removal and nocturnal sedation are two possible examples given in the UK GPG [21].

**Table 6:** Percentage of patients classified as having tinnitus symptoms that are not compensated or compensated, split by each country, and reported separately for each professional group.

	GP profession		ENT profession	
**Country**	**Not compensated [%]**	**Compensated** **[%]**	**Not compensated [%]**	**Compensated** **[%]**
				
US	34	66	26	74
UK	41	59	35	65
Germany	47	53	30	70
France	49	51	35	65
Spain	39	61	39	61
Italy	40	60	42	58

Data are reported only for those physicians who saw at least one tinnitus patient in the past three months.

### Referral to GPs and ENTs

For this question in the survey (Q3, Appendix 1), respondents were asked to consider their last 10 patients with subjective tinnitus. Responses tell us about the proportion of those patients who directly accessed the service and those who were referred from elsewhere. Reports are summarized in Table 7 as percentages. In the case of GPs, almost all tinnitus patients (>90%) came to the surgery directly (without a referral). As expected, the proportion of 'direct access' patients was somewhat lower for ENTs (45-60% in all countries except Italy), with some apparent variation across countries. For example, in the UK only 14% of tinnitus cases in ENT surgeries were direct access, while in Italy this estimate reached 68%. This finding is most likely to reflect differences in the healthcare systems. In the UK for example, direct access to specialist services is rarely an option for patients [25].

**Table 7:** Percentage of patients with subjective tinnitus who directly accessed the consultation with the physician and those who were referred by another healthcare professional, split by each country, and reported separately for each professional group.

	GP profession		ENT profession	
**Country**	**Direct** **[%]**	**Referred[%]**	**Direct** **[%]**	**Referred** **[%]**
				
US	94	6	45	55
UK	98	2	14	86
Germany	94	6	55	45
France	96	4	57	43
Spain	93	7	60	40
Italy	95	5	68	32

Data are reported only for those physicians who saw at least one tinnitus patient in the past three months.

The physicians were also asked about the type of healthcare professionals who referred cases of subjective tinnitus to them (Q4, Appendix 1). A range of options included GPs, other ENTs, neurologists, psychotherapists or specialists in psychosomatic disorders, neurosurgeons, radiologists, audiologists and paediatricians. Responses from GPs on this question are not so informative since such a large number of cases are direct access. For ENTs with referred patients, at least 89% in all countries reported that GPs referred patients to them. Audiologists seemed to play an important role in those countries where audiology is a recognized profession in its own right, with 55% of UK ENTs stating receiving such referrals, and 32% of US ENTs. In all countries except France (6%), ENTs also listed neurologists as another key source of referrals (>20%), with this being particularly high in Germany (44%). This may be related to the fact that in Germany many neurologists are both neurologists and psychiatrists.

### Diagnosis and onward referrals

Table 8 reports percentages of reported patients who were diagnosed as having tinnitus by another specialist before reaching the respondent, those who were diagnosed by the physician themselves and those who needed to be referred on for diagnosis (Q5 and Q6, Appendix 1).

**Table 8:** Percentage of patients reported to have been diagnosed as having tinnitus by another specialist before reaching the respondent ('pre-diagnosed'), diagnosed by the physician themselves ('Self') or needing to be referred on for diagnosis ('Referred on').

	GP profession			ENT profession		
**Country**	**Pre-diagnosed [%]**	**Self** **[%]**	**Referred on** **[%]**	**Pre-diagnosed [%]**	**Self [%]**	**Referred on [%]**
						
US	13	62	25	24	71	5
UK	14	63	23	**31**	63	6
Germany	20	37	43	23	68	9
France	15	66	18	35	61	4
Spain	14	59	26	22	74	4
Italy	11	54	35	28	63	10

Data are split by each country, and reported separately for each professional group. Again, only data for those physicians who saw at least one tinnitus patient in the past three months are reported.

The proportion of tinnitus cases diagnosed by the GP themselves was almost 60% in all countries except Germany (37%). The proportion patients with a 'pre-diagnosis' was small in GP surgeries (11-20%), while the number of patients who were referred by a GP on to a colleague for a diagnosis accounted for about 25% in the US, UK and Spain, and slightly less in France (18%). Particularly in Germany, but also in Italy, considerably more patients were referred for diagnosis (43% and 35% respectively). These numbers may reflect easier access to relevant specialist care in these two countries.

The physicians were asked about the type of healthcare professionals they referred their patients to for diagnosis. Predominantly such a colleague was judged to be an ENT specialist (>90% of GPs referring patients for diagnosis reported to do so to an ENT specialist). This pattern is possibly indicative of the dominance of otolaryngology as the medical specialty for hearing-related disorders (see Discussion for further details). In Germany and Italy, where a smaller percentage of GPs diagnosed tinnitus themselves, GPs typically chose to refer on to an ENT doctor and a neurologist (Table 8). In Germany, estimates indicated 100% of onwards referrals were to ENT with 46% also to a neurologist. Similarly in Italy, 92% of onward referrals were judged to be to ENT with 38% also to neurology.

ENTs estimated that they diagnosed about the same proportion of tinnitus cases themselves as did GPs (61-74%). Generally, ENTs received more pre-diagnosed cases (about 27%) than did GPs, while a smaller proportion (4-10%) was referred elsewhere. Such onward referrals were made to a variety of healthcare professionals including neurologists, radiologists, and audiologists. It is interesting to note that in the UK, none of the ENT respondents reported making a referral to neurology, while in all other countries this accounted for 32-60% of such onward referrals, even in the US. According to the responses, the patterns reported for ENT were more consistent between the other four European countries.

### Management and onward referrals for management

The questionnaire asked about patients with subjective tinnitus who were managed by the respondent and those who were referred on for management (Q7, Appendix 1). Reports are summarized in Table 9.

**Table 9:** Percentage of patients with subjective tinnitus that are managed by the physician themselves ('Self') or are referred on for management by another specialist ('Referred on'), split by each country, and reported separately for each professional group.

	GP profession		ENT profession	
**Country**	**Self** **[%]**	**Referred on [%]**	**Self** **[%]**	**Referred on [%]**
				
US	67	33	94	6
UK	67	33	79	21
Germany	69	31	92	8
France	71	29	92	8
Spain	69	31	96	4
Italy	58	42	90	10

Data are reported only for those physicians who saw at least one tinnitus patient in the past three months.

GPs in all countries stated that around two thirds of patients (58-71%) were managed by themselves. Of the remaining patients that were referred on for management, it was mainly to ENTs (stated by ≥89% of GPs), which agrees internally with the information given by ENTs about the source of their referrals. It is interesting to note that a recent independent survey conducted in England found that GPs referred on 37% of tinnitus patients, most of which were either to ENT (82%) or audiology (12%) [18]. These values provide some external corroboration for the UK-bases estimate in the present study where 33% of tinnitus patients were referred on by GPs. The proportion of tinnitus patients who were treated by ENTs themselves was at least 90% in each country, except in the UK (79%). Here, of the remaining 21% that were referred on, it was mostly to audiologists (stated by 68% of ENTs), psychotherapists (13%), and hearing therapists (10%).

In summary, our findings highlight the important role of GPs and ENTs in the diagnosis and management of tinnitus across all six countries. In those countries surveyed, GPs and ENTs played a frequent role in the diagnosis and management of tinnitus, while such a role for other professions such as neurology, radiology and psychology differed across countries. In Germany, neurology was the predominant 'external' speciality with the largest proportion of tinnitus cases, with Italy and Spain following behind. In these countries, neurologists seem to play an important role in both diagnosis and management. Interestingly, neurology was never the choice for diagnosis or management for UK ENT respondents (9% of UK GPs referred patients to neurologists for management). Instead in the UK, audiology dealt with the greatest number of tinnitus referrals by ENTs (68%) with 36% of US-based ENTs also following the same commissioning route. This is consistent with the recognition of audiology as an independent profession in these two countries (see Discussion). In all six countries, ENTs occasionally mentioned radiologists, but this was primarily in a diagnostic role, not for management. For example, the TRI guidelines recommend angiography, magnetic resonance imaging or echo-doppler testing in cases of pulsatile tinnitus that is suspected to be associated with a non-otological cardiovascular condition. In the UK, tinnitus with sudden or rapidly progressive hearing loss is a 'red flag' for immediate referral to another medical discipline, such as radiology [21].

The physicians were asked about treatments normally offered to people with subjective tinnitus (Q9, Appendix 1). Respondents chose from 10 options, with no restriction on the number of options that could be selected. For clarity, treatment options for acute and chronic tinnitus are reported separately.

### Typical treatment options for acute tinnitus

**Figure 1:**
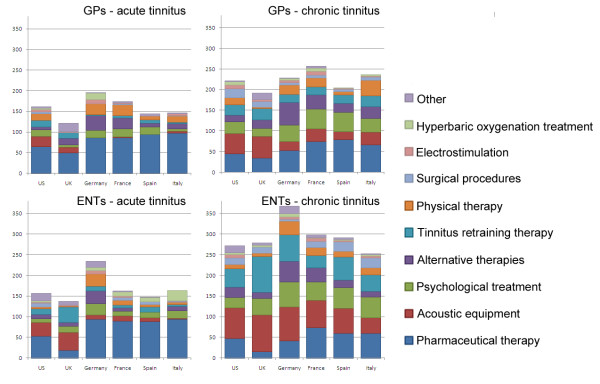
**Number of physicians reporting the availability of a range of normal treatment options for acute and chronic tinnitus, reported separately by GPs and ENTs**.

About half of all responding GPs reported the use of a pharmaceutical treatment as a course of treatment for acute tinnitus (average = 77.1%). GPs in Italy and Spain reported the highest usage of medication as a treatment option (98% and 94%, respectively), with the UK (49%) and US (64%) reporting the least usage. The next most popular form of treatment offered in all countries was some form of alternative therapy (average = 16.1%), used most widely in Germany (36%). Other treatments were used to a lesser extent and showed some degree of preference by country. Psychological treatment was most common in France, Spain, Germany, and the US (16-20%), while GPs in the UK and the US tended to offer forms of tinnitus retraining therapy (14% and 16%, respectively). In the UK, there is a severe shortage of qualified clinical psychologists and psychotherapists for tinnitus counselling, and so GPs are advised [21] to provide 'informational counselling' which is based on technical education and information giving. Audiology departments in the UK are expected to provide some form of counselling, but this more typically relates to personal adjustment counselling as part of the audiological rehabilitation which is targeted towards helping patients to confront a range of psychological, social and emotional concerns as they relate to audiology. Acoustic devices were reported to be offered to patients with acute tinnitus in the US (25%) and in the UK (14%), probably reflecting the recognized status of the audiological profession in these healthcare systems. Physical therapy was most prevalent in France, Germany, the US and Italy (15-26%). This type of therapy mobilizes joints and massages soft tissue. GPs in Germany were noted as the only primary-care practitioners to substantially refer patients to hyperbaric oxygenation treatment (16%), which delivers oxygen to the patient at a level higher than atmospheric pressure. These findings are illustrated in Figure 1 (top left hand panel).

**Figure 2:**
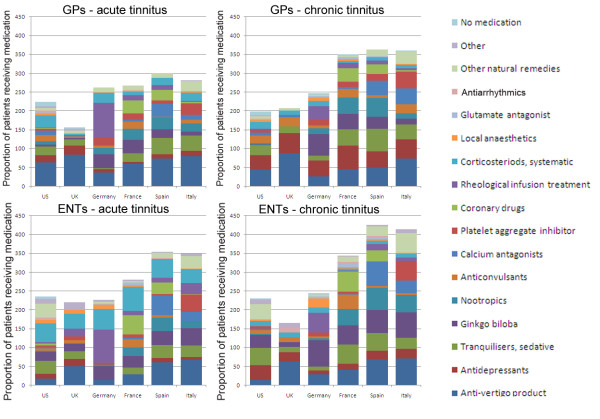
**Proportion of patients (%) offered particular pharmaceutical treatments as a first line approach for acute and chronic tinnitus, reported separately by GPs and ENTs**.

Again, most responding ENTs reported pharmaceutical treatments for acute tinnitus (average = 69.9%), although the UK was considerably lower than this average (19%) (see Figure 2, bottom left hand panel). Instead in the UK, acoustic equipment (43%) and tinnitus retraining therapy (38%) were the most preferred treatment options, with possible reasons for this pattern having already been presented above. Acoustic devices were also a common option for ENTs in the US (33%) and in the UK (43%). In Germany, the low numbers of ENTs considering acoustic treatments (10%) is probably indicative of a healthcare system in which otolaryngologists in private practice and in hospitals do not stock hearing aids [26]. ENTs practicing in Italy were more likely to offer hyperbaric oxygenation treatment (25%) than ENTs elsewhere, while physical therapy was popular in German ENT clinics (30%).

### Typical treatment options for chronic tinnitus

GPs in all six countries estimated that they used pharmaceutical therapies considerably less often for chronic tinnitus than they did for acute tinnitus (average = 55.8%) (Figure 1, top right hand panel). France and Spain were those countries with the highest proportion of drug treatments (74% and 78% respectively), with Italy (66%) and Germany (52%) trailing. For chronic tinnitus however, acoustic devices (average = 36.3%) and psychological treatment (average = 35.1%) were commonly offered by GPs in all six countries, with alternative therapies the next most popular option (average = 26.6%). Physical therapy stood out as one of the most popular options in Italy (38%), behind that of a pharmaceutical approach.

For ENTs, there was again a reduction in the prevalence of pharmaceutical medication in cases of chronic tinnitus (Figure 1, bottom right hand panel) compared with acute tinnitus, particularly in Germany. In the UK, drugs were rarely offered (15%). Across all countries, the preference of ENTs offering acoustic equipment, psychological treatment and tinnitus retraining therapy to tinnitus patients was greater for chronic cases (averages = 68.8%, 42.3% and 52.4%, respectively) than for acute cases (averages = 20.6%, 15.0% and 14.5%, respectively). Alternative therapy also counted for a substantial proportion of the available treatment options for chronic tinnitus (average = 25.9%) and physical therapy was again popular in Germany (34%).

One general interpretation of the data presented in Figure 1 is that the range of treatments offered by an individual physician is broader in scope for chronic tinnitus than for acute tinnitus. This is evidenced by the height of the bars in each panel.

Furthermore, the questionnaire asked about first-line treatment options for patients with acute and chronic subjective tinnitus (Q10, Appendix 1). Broadly speaking, the patterns emerging were equivalent to the results reported above for the previous question. However, responses to this question gave additional information about the proportion of patients not treated and results showed that about 21% of cases did not receive any specific treatment. This proportion did not markedly differ across professions (i.e. GP vs ENT) or pathology (i.e. acute vs chronic tinnitus). However, several country-specific patterns are noteworthy. In Germany, very few acute cases remained untreated by GPs (12%) and ENTs (2%). In contrast, in the UK, a relatively high proportion of acute cases were reported not to be offered a first-line treatment (GPs = 44% and ENTs = 54%).

Another question asked about first-line pharmaceutical treatments for patients with acute and chronic subjective tinnitus (Q11, Appendix 1). Respondents chose from 17 options, with no restriction on the number of options that could be selected. Again, for clarity, treatment options for acute and chronic tinnitus are reported separately.

### Pharmaceutical treatments used as a first-line approach for acute tinnitus

Rather broad diversity in drug treatments was noted for acute tinnitus (Figure 2, left hand panels). However GPs tended to prefer anti-vertigo products in all countries (average = 65.4%), except in Germany (37%). Here rheological infusion treatment dominated as the medical approach of first choice (93%). We have already noted that rheological infusion was little reported elsewhere. Other features of GP practice in Germany were the preference for ginkgo preparations (37%) and corticosteroids (26%). Ginkgo biloba was also often used in France (35%), while corticosteroids were also common as a first-line treatment in the US (31%). Again, these drugs were prescribed less frequently elsewhere. In Italy and Spain, tranquillisers were often prescribed for acute forms of tinnitus (41% and 43%, respectively). In Spain and France, nootropics and coronary drugs accounted for 30-32% of the drugs recommended by GPs for acute tinnitus, while in Italy platelet aggregation inhibitors accounted for about 31% of the drugs used in that country. These drugs are effective for preventing arterial thrombosis.

Results for ENTs are shown in the bottom left hand panel of Figure 2. Among ENTs in all countries, anti-vertigo products were mentioned considerably less often (average = 37.5%) than in GP practice, and in Germany these accounted for only 13% of all drugs recommended by ENTs for acute tinnitus. Like GPs in Germany, ENT specialists also favoured rheological infusion (89% of all drug treatments). Also of note was the greater use of corticosteroids by ENTs in all six countries (average = 50.6%) compared to GPs (average = 20.6%). Ginkgo biloba remained a popular first line choice (average = 34.2%), except in the UK (20%). In Spain, calcium antagonists accounted for 52% of drug treatments, while ENTs in France used coronary drugs most often (51%). In Italy, ENTs (like their GP counterparts) preferred the use of platelet aggregation inhibitors (45%) as one of the first-line pharmaceutical options.

### Pharmaceutical treatments used as a first-line approach for chronic tinnitus

Once again, very broad diversity in drug treatments was noted for chronic tinnitus (Figure 2, right hand panels). Reports by GPs regarding chronic tinnitus are shown in the top right hand panel of Figure 2. For chronic tinnitus, anti-vertigo products were widely used by GPs (average = 51.8%), but not as often as for acute tinnitus. According to the data, patients visiting a GP in the UK were most likely to be prescribed an anti-vertigo product (acute tinnitus = 84% and chronic tinnitus = 88%). The second most popular first-line treatment by GPs for chronic tinnitus was an anti-depressant (average = 47.7%). As for acute tinnitus, pharmaceutical treatments differed somewhat across countries. Tranquilisers were common in Spain, the US, France and Italy (59%, 27%, 43% and 39%, respectively) and this pattern was broadly repeated by ENTs for patients with chronic tinnitus too. Corticosteroids were again used most often by GPs practicing in the US (18%) and in Germany (26%), but less commonly than for acute tinnitus. Also in Germany was the continued GP preference for ginkgo preparations (58%), but while rheological infusion had been the dominant treatment for acute tinnitus, it accounted for only 35% of treatments offered to patients with chronic tinnitus.

Reports by ENTs regarding chronic tinnitus are shown in the bottom right hand panel of Figure 2. ENTs appeared to be as likely to prescribe anti-vertigo products for chronic tinnitus (average = 42.6%) as they would for acute tinnitus. Again, patients visiting ENT in the UK would most likely be prescribed an anti-vertigo product (chronic tinnitus = 63% and acute tinnitus = 50%) compared to any other type of drug. The second most popular first line treatment by ENTs for chronic tinnitus was Ginkgo biloba (average = 52.3%), particularly in Germany (71%). Again, this repeats a pattern observed for ENT treatment of acute tinnitus. Anti-depressants and tranquilisers were also prescribed for tinnitus in all six countries (average = 25.0% and 38.6%, respectively), but to a somewhat lesser degree in Germany. Overall, corticosteroids accounted for fewer first-line drug treatment options in cases of chronic tinnitus (average = 10.8%), than acute tinnitus.

A number of country-specific preferences prevailed. For example, in Spain, calcium antagonists accounted for 65% of drug treatments, while ENTs in France used coronary drugs most often (54%). In Italy, ENTs (like their GP counterparts) preferred platelet aggregation inhibitors (52%) as one of their first-line drug options. Nootropic preparations were popular in Spain (58%), France (43%) and Italy (45%), but were seldom used elsewhere. Finally, rheological infusions in Germany accounted for 52% of first line drug options.

For second-line medication options, the overall picture was not well differentiated and so responses to the corresponding question (Q12) are not reported.

### Satisfaction with medication options for subjective tinnitus

The physicians were asked to rate their satisfaction with current medications for treating subjective tinnitus on a 4-point scale and give reasons for selecting that answer (Q13 and Q14, Appendix 1). GPs and ENTs responded similarly with most of them (>60%) being dissatisfied with current drug treatments (Table 10). There were no striking differences of opinion across the six countries, with the most common reason being a general lack of effectiveness. In Germany, a large number of GPs and ENTs also reported a high relapse rate (44% and 36%, respectively).

**Table 10:** Percentages of satisfaction ratings by physicians regarding current medications for subjective tinnitus.

	Very satisfied [%]		Relatively satisfied [%]		Not very satisfied [%]		Completely dissatisfied [%]	
**Country**	**GP**	**ENT**	**GP**	**ENT**	**GP**	**ENT**	**GP**	**ENT**
								
US	5	3	34	14	57	60	4	24
UK	2	2	12	23	61	40	25	36
Germany	0	2	28	26	50	60	22	12
France	0	0	18	12	64	70	18	18
Spain	0	4	38	23	56	58	6	15
Italy	2	2	34	29	58	60	6	10

Ratings reported by each profession each sum to 100%.

Data are reported only for those physicians who saw at least one tinnitus patient in the past three months.

### Success rate of typical treatment options

The physicians were asked to estimate what proportion of treatments was successful for patients with subjective tinnitus (Q15, Appendix 1). For chronic tinnitus, GPs and ENTs rated treatments as being successful in approximately one third of all cases (22-37% and 20-57%, respectively). Success rate was judged to be somewhat better for acute tinnitus (GPs: 37-51% and ENTs: 43-61%). While this pattern was shared among most countries, in the UK ENTs were markedly more positive and perceived success in about 10% more cases of chronic and acute tinnitus than the average.

### Perceived challenges in treating subjective tinnitus

The questionnaire included a request to highlight any problems in treating subjective tinnitus using current standards of practice (Q16, Appendix 1). Respondents from all six countries touched on the theme of effectiveness (GPs: 37-60%; ENTs: 36-55%), highlighting a low success rate as being a key problem. A range of other issues were also raised, but these differed somewhat from country to country and across the two professions. For example, in France, a quarter of GPs were concerned about the role of the patient's psychological distress (24%), while one third of ENTs were concerned about a lack of focus on treatment for depression (32%). In Italy, many ENTs expressed patient's resistance to therapy as problematic (27%), while in the US, the shared concern for ENTs was the lack of possibilities to treat the cause of tinnitus (23%).

## Discussion

The present survey paints a picture in which the characteristics of tinnitus (i.e. pathogenesis, duration and severity) in those people seeking professional help are perceived to be broadly the same across the six countries. The data indicate that the 'most typical' sort of tinnitus patient seeking a consultation with their GP tends to be someone with chronic subjective tinnitus who has a repeat appointment. ENTs see many more patients with tinnitus and these tend to be first-time appointments, but again with chronic subjective tinnitus as the defining feature. Despite these commonalities, clinical provision differs greatly across countries; not only in terms of their journey through the healthcare system, but also in terms of the most likely options offered to them for treating their tinnitus. In this Discussion, we summarize some of these issues emerging from the present self-report data and speculate on the key drivers behind those differences.

### Principal differences in the roles of GPs and ENTs

Regarding treatment of chronic tinnitus, we observed highly varying approaches, across professions and also across countries. GPs are often the 'first port of call' for a patient seeking help with their tinnitus, while most ENTs see referred patients, typically from GPs, but also from audiologists (UK) and neurologists (Germany). Typically, GPs and ENTs reported that they were responsible for diagnosing tinnitus in about two-thirds of all cases. According to reports, there was a differing level of involvement in tinnitus management. GPs in all countries stated that around two thirds of patients were managed by themselves, whereas a higher proportion of tinnitus patients were reportedly treated by ENTs themselves. The survey highlighted the role of other professions in several European countries in terms of a role in management. Notably this included specialists in neurology in Germany, Italy and Spain and audiologists in the UK and US. The involvement of neurologists may reflect increasing awareness of the role of the central nervous system for tinnitus generation and maintenance by GPs and ENT specialists. In the following section, we describe differences in the provision of hearing healthcare specialties which go some way to address the differing roles of the audiology profession.

### Specialized clinical training in hearing problems

The healthcare system would appear to strongly determine how specialist treatment for tinnitus is provided. In this section, we consider the training routes and professional recognition of ENT and audiology specialties across Europe and the US in order to contextualize some of our findings, Otorhinolaryngology represents an established area of health care for disorders of the head and neck, including hearing, balance, sinus, nose, voice, oral cavity, sleep, allergy, and head and neck tumors. Within Europe, the European Union of Medical Specialists (UEMS), founded in 1958, has ensured the quality and harmonization of specialist training in order to support the free movement of qualified physicians and patients. In otorhinolaryngology, this has been achieved through a Charter in 1995 and a European training programme in 2009 for head and neck surgery and ENT specialists http://www.orluems.com/index.asp. In the USA, the Accreditation Council for Graduate Medical Education achieves similar aims, with 105 programs in otolaryngology (2011-12) achieving national standards in medical training http://www.acgme.org/adspublic/reports/accredited_programs.asp.

In contrast, audiology represents a younger area of health care for hearing and balance disorders, with specialist skills in rehabilitation especially in the provision of hearing aids. In the US, audiology is recognized as an independent profession in its own right, and training and practice are well regulated. In Europe however, the situation is considerably more variable. Although the profession is represented by the European Federation of Audiology Societies (EFAS), founded in 1992, there is no Europe-wide standard for training or professional recognition. Specialist training in the UK and Scandanavia has been well established for many years and EFAS supports a proposal for a 'General Audiologist profession' to standardize the training curriculum and professional skills more widely. Training provision is growing in some countries (e.g. Spain now offers two Masters in Audiology courses on this model). However, it remains unclear whether the structure and status of the audiology profession can ever be consistent throughout Europe, given relative strength of the medical professions in certain countries. For example, in Spain, audiology is not an official profession, although hearing-aid technicians were officially recognized in 2001.

It is interesting to note that in Europe other professions lay 'claim' to audiology and the EU perspective from UEMS is one of audiology as a sub-speciality of ENT. In Italy, audiology was previously a speciality within ENT, but is now divided between ENT and phoniatrics (i.e., speech and language assessment and treatment) (Ferdinando Grandori, personal communication). In Germany, ENTs deal with differential diagnostics and management of hearing loss, a second, independent ENT specialty combines phoniatrics and pediatric audiology [26] and hearing aid fitting is provided by the independent speciality of hearing-aid technicians. Of the five European countries surveyed here, the UK is unusual in that audiology is a recognized specialism in its own right and as a consequence is the primary health system provider of treatments for chronic tinnitus [25]. In this respect, the UK system is more similar to that of the US, than its European neighbours.

Training routes and recognized specialisms are therefore highly likely to contribute to the variability in clinical practice across the different countries.

### Management of acute subjective tinnitus: Key differences across Europe and the US

A substantial proportion of responding GPs and ENTs employed pharmaceutical treatment as a course of treatment for acute subjective tinnitus, but countries appear to differ in their use of drugs as a *typical *course of treatment for acute tinnitus. Broadly speaking, the same patterns of treatment for acute tinnitus were reported by GPs and ENTs. Italy and Spain appear to use medications most frequently, with the UK and US reporting the least usage. The survey findings indicate treatment with anti-vertigo products or corticosteroids are widely accepted options. Treatments involving some form of psychological approach (including TRT) were most common in France, Germany, UK and the US, while the UK and US also offered acoustic devices. Physical therapy was most prevalent in France, Germany, the US and Italy. GPs in Germany were noted as the only primary-care practitioners to substantially offer rheological infusion and hyperbaric oxygenation treatments. In the absence of any robust evidence base for the efficacy of drug treatments for subjective tinnitus, one might draw on anecdotal observations to begin to understand these differences. For example, it is interesting to note that in Germany, specialist hyperbaric oxygen therapy centres have widely advertised the benefits of this therapy for tinnitus perhaps thus increasing awareness in the clinical profession and rheological infusion was covered by medical insurance at the time of the online survey. These social and political contextual factors might exert strong influences on healthcare decisions. Taking the US as a comparison example where hyperbaric oxygen therapy was rarely used, although some US alternative physicians have extended the use of this treatment to include tinnitus, to our knowledge tinnitus is not an indication that is approved by the UHMS Hyperbaric Oxygen Therapy Committee nor is it reimbursed by US medical insurance (e.g. Medicare).

By way of minor comment we reflect on the possibility that not all respondents approached this question from the point of view of what *they themselves *would offer, but instead responded according to what the *local healthcare *system would typically offer. Evidence to support this view comes from the pattern of UK responses, since acoustic devices are always provided by audiology services, not by GPs and ENTs.

### Management of chronic subjective tinnitus: Key differences across Europe and the US

A large number of patients develop chronic tinnitus and so the greater challenge remains the management of this long-lasting condition. Pharmacological interventions appear to be less well regarded as effective for chronic forms of tinnitus than for acute forms of the condition, both by GPs and ENTs. Nevertheless, in Italy and Spain medications were again prescribed more frequently than elsewhere. Instead, a general trend was observed for a greater usage of acoustic instruments and psychological approaches. In addition to these conventional therapies, clinicians were more likely to offer a broad range of approaches the choice of which appeared to be more driven by the country in which the clinician was based than the form of subjective tinnitus being presented. Again this suggests social and political factors are more influential than clinical evidence regarding efficacy.

### Low satisfaction with available treatment options

This was unequivocally mentioned by both GPs and ENTs from all investigated countries. Thus clinical experiences under real-world conditions are in line with systematic reviews and meta-analyses of controlled studies indicating low evidence for the efficacy of the various treatment options for tinnitus [14,16,27].

## Conclusions

From the sample of physicians participating in this survey, a wide variety of treatment approaches (both pharmaceutical and non-pharmaceutical) appear to be employed by GPs and ENTs across Europe and the US, albeit all with rather poor patient outcomes. While some commonalities in treatment approach have been observed, substantial differences were noted across the six countries, in part but not always related to the national healthcare system. Healthcare professionals from all six countries express their dissatisfaction with current practice and the results of this survey highlight the need for an effective therapy option, particularly for chronic subjective tinnitus, with guidelines about how to use diagnostic criteria to guide prescription

## Competing interests

The research was commissioned by Merz Pharmaceuticals GmbH.

## Authors' contributions

MK defined the research theme, contributed to study design, data collection and discussion of the analysis. DAH provided interpretation of the data, drafted the manuscript and prepared the figures. MJAL, CWN, TGS, ME, FT and BL critically reviewed the data interpretation and the manuscript. All authors have contributed to, seen and approved the manuscript.

## Appendix 1: Survey questions

Q1. How many patients with tinnitus problems in total did you see in the last three months? And how many new patients with tinnitus have come to you for treatment within the last three months?

Q2. Tinnitus can be classified according to different criteria, such as its pathogenesis (subjective vs. objective), its duration (acute vs. chronic) or the perceived severity of the disorder (compensated vs. not compensated). If you consider the cases that you saw in the last three months, how could they be classified according to these criteria?

Q3. Please consider your last 10 patients with subjective tinnitus. How many of these patients came to your practice directly (without a referral) and how many were referred to you by other doctors/specialists?

Q4. Which doctors/specialists made this referral?

Q5. Please think again about your last 10 patients with subjective tinnitus in terms of their diagnosis. How many of them did you diagnose yourself, how many were already pre-diagnosed by another doctor and how many did you refer to other specialists for further diagnostic confirmation?

Q6. You have just indicated that you referred of your tinnitus patients to another specialist for diagnostics. Who were these specialists?

Q7. Please think again about your last 10 patients with subjective tinnitus in terms of their management. How many of them did you manage yourself and how many did you refer to other specialists for further management?

Q8. You have just indicated that you referred of your tinnitus patients to another specialist for further management. Who were these specialists?

Q9. Which course of treatment do you normally use for patients with tinnitus? Please distinguish between acute and chronic forms of tinnitus.

Select from list

Other (please give details)

Q10. Please consider your first-line treatment choices. If you now think of your last 10 patients with acute subjective tinnitus and your last 10 patients with chronic subjective tinnitus: How would you categorise them in the following courses of treatment?

Select from list

Other (please give details)

Patients not treated

Q11. If you treat subjective tinnitus with medication, which would be your first-line treatment?

Select from list

Other (please give details)

No medication

Q12. Which medications do you use as second choice, if your first line treatment is unsuccessful?

Select from list

Other (please give details)

No medication

Q13. How satisfied are you overall with current medications for treating subjective tinnitus?

Very satisfied

Relatively satisfied

Not very satisfied

Completely dissatisfied

Q14. You have just indicated how satisfied you are with current medications for treating subjective tinnitus. Please give your reasons for choosing that answer in as much detail as possible.

Q15. If you consider all of your patients with subjective tinnitus (both acute and chronic and regardless of whether medication is used or not) for what proportion of them do you believe that the treatment used has been successful?

Q16. Are there any problems/difficulties with treating subjective tinnitus that you are unable to solve by means of the current standards of practice? If so, what are these problems/difficulties in the management of subjective tinnitus?

## Pre-publication history

The pre-publication history for this paper can be accessed here:

http://www.biomedcentral.com/1472-6963/11/302/prepub

## References

[B1] Eggermont JJ Roberts LE The neuroscience of tinnitus Trends Neurosci 2004 27 11 676 682 10.1016/j.tins.2004.08.010 15474168

[B2] Møller AR Tinnitus: Presence and future Prog Brain Res 2007 166 3 16 17956767 10.1016/S0079-6123(07)66001-4

[B3] Davis AC Hearing in adults 1995 London: Whurr Publishers

[B4] Davis A El Rafaie A Tyler RS Epidemiology of tinnitus Tinnitus Handbook 2000 Singular, Thomson Learning: San Diego 1 23

[B5] Ahmad N Seidman M Tinnitus in the older adult: Epidemiology, pathophysiology and treatment options Drugs Aging 2004 21 5 297 305 10.2165/00002512-200421050-00002 15040757

[B6] Erlandsson SI Hallberg LR Prediction of quality of life in patients with tinnitus Br J Audiol 2000 34 1 11 20 10.3109/03005364000000114 10759074

[B7] Bauch CD Lynn SG Williams DE Mellon MW Weaver AL Tinnitus impact: Three different measurement tools Journal of the American Academy of Audiology 2003 14 4 181 187 12940702

[B8] Hallam RS Jakes SC Hinchcliffe R Cognitive variables in tinnitus annoyance Br l Clin Psychol 1988 27 3 213 222 10.1111/j.2044-8260.1988.tb00778.x 3191301

[B9] Axelsson A Prasher D Tinnitus induced by occupational and leisure noise Noise Health 2000 2 8 47 54 12689461

[B10] Vernon JA Møller AR Mechanisms of tinnitus 1995 Needham Heights, MA: Allyn and Bacon

[B11] Henry JA Dennis KC Schechter MA General review of tinnitus: prevalence, mechanisms, effects, and management J Speech Lang Hear Res 2005 48 5 1204 1235 10.1044/1092-4388(2005/084) 16411806

[B12] Adjamian P Sereda M Hall DA The mechanisms of tinnitus: Perspectives from human functional neuroimaging Hearing Research 2009 253 15 31 10.1016/j.heares.2009.04.001 19364527

[B13] Vio MM Holme RH Hearing loss and tinnitus: 250 million people and a US$10 billion potential market Drug Discov Today 2005 10 19 1263 1265 10.1016/S1359-6446(05)03594-4 16214667

[B14] Langguth B Salvi R Elgoyhen AB Emerging pharmacotherapy of tinnitus Expert Opinion on Emerging Drugs 2009 14 687 702 10.1517/14728210903206975 19712015 PMC2832848

[B15] Noble W Treatments for Tinnitus Trends in Amplification 2008 12 3 236 241 10.1177/1084713808320552 18635586 PMC4134891

[B16] Dobie RA Snow JB Junior Clinical trials and drug therapy for tinnitus Tinnitus: Theory and management 2004 Lewiston, NY: BC Decker 266 277

[B17] Vanniasegaram I Cadge B McKenna L Hinchcliffe R A postal survey of tinnitus management in general practice Journal of Audiological Medicine 1993 2 1 8

[B18] El-Shunnar S Hoare DJ Smith S Gander PE Kang S Fackrell K Hall DA Primary care for tinnitus: practice and opinion among GPs in England Journal of Evaluation in Clinical Practice 2011 17 4 684 92 10.1111/j.1365-2753.2011.01696.x 21707872 PMC3170708

[B19] Naughton P The quest for quiet: People's experience of tinnitus in Ireland 2004 Dublin: Irish Tinnitus Association

[B20] Redmond S What's that noise? A profile of personal and professional experience of tinnitus in Northern Ireland 2010 Belfast: Royal National Institute for Deaf People

[B21] Department of Health (UK) Improving Access to Audiology Services in England London 2007 http://www.dh.gov.uk/en/Publicationsandstatistics/Publications/PublicationsPolicyAndGuidance/DH_093844

[B22] Biesinger E Del Bo L De Ridder D Goodey R Herraiz C Kleinjung T Lainez JM Landgrebe M Langguth B Londero A Paolino M Questier B Sanchez T Searchfield G Algorithm for the diagnostic and therapeutic management of tinnitus 2010

[B23] http://www.tinnitusresearch.org/en/documents/downloads/TRI_Tinnitus_Flowchart.pdf

[B24] Hoare D Hall DA Clinical guidelines and practice: a commentary on the complexity of tinnitus management Health and the Health Professions 2011 in press 10.1177/0163278710390355 PMC3757916 21177640

[B25] Hoare DJ Gander PE Collins L Smith S Hall DA Management of tinnitus in English NHS Audiology Departments: an evaluation of current practice Journal of Evaluation in Current Practice 2011 17 10.1111/j.1365-2753.2010.01566.x PMC3489049 21087449

[B26] Gander PE Hoare DJ Collins L Smith S Hall DA Referral pathways for tinnitus management: a comprehensive survey of NHS Audiology Departments in England BMC Health Services Research 2011 11 162 10.1186/1472-6963-11-162 21733188 PMC3144449

[B27] Lenarz T Ernst A Medical Audiology in Germany: before and since reunification American Journal of Audiology 1995 4 1 9 11

[B28] Hoare DJ Kang S Hall DA Systematic review and meta-analyses of RCTs examining tinnitus management The Laryngoscope 2011 121 7 1555 1564 10.1002/lary.21825 21671234 PMC3477633

